# On the intensity decay of tropical cyclones before landfall

**DOI:** 10.1038/s41598-022-07310-4

**Published:** 2022-02-28

**Authors:** S. Wang, R. Toumi

**Affiliations:** Department of Physics, Imperial College London, London, SW7 2AZ UK

**Keywords:** Atmospheric science, Natural hazards

## Abstract

It remains unclear how tropical cyclones (TCs) decay from their ocean lifetime maximum intensity (LMI) to landfall intensity (LI), yet this stage is of fundamental importance governing the socio-economic impact of TCs. Here we show that TCs decay on average by 25% from LMI to LI. A logistic decay model of energy production by ocean enthalpy input and surface dissipation by frictional drag, can physically connect the LMI to LI. The logistic model fits the observed intensity decay as well as an empirically exponential decay does, but with a clear physical foundation. The distance between locations of LMI and TC landfall is found to dominate the variability of the decay from the LMI to LI, whereas environmental conditions are generally less important. A major TC at landfall typically has a very large LMI close to land. The LMI depends on the heating by ocean warming, but the LMI location is also important to future landfall TC intensity changes which are of socio-economic importance.

Tropical cyclones (TCs) are one of the major global natural hazards and of great concern to coastal regions. The TC intensity is traditionally recorded as the surface maximum sustained wind. The lifetime maximum intensity (LMI) is the intensity record that has been extensively studied. The LMI is the intensity that is closest to a theoretical upper limit, i.e., the maximum potential intensity, which can be predicted based on environmental thermodynamic conditions^1,2^. Previous studies^3–5^ showed that the LMI has been increasing in the past decades. The location of LMI is also migrating toward the coasts^6^ and the poles^7^, potentially due to the expansion of the tropics^8^. Regionally, this coastal poleward shift of LMI may change the TC threat to the coasts in the western North Pacific^9–11^ and elsewhere. However, the location of LMI is on average more than 700 km to the coastline^6^ and this distance is much larger than the typical TC wind radius of gale-force wind of about 200 km^12^.

It is the landfall intensity (LI) that dominates the destructive potential in coastal regions^13,14^. More than 80% of normalized TC-related damage in the US is caused by the landfall TCs with major hurricane intensities, that is, LI ≥ 50 m s^−1^. The intensity from the LMI to landfall have a wide range of behaviours. For example, Hurricane Dorian (2019) made landfall in the Bahamas just after a raid intensification^15^. However, Typhoon Kong-rey (2018) decayed from a category-5 intensity by about 50% to landfall in South Korea^16^. To date, there has been no systematic analysis of the intensity decay from the LMI to LI, which we provide here.

Internal and environmental factors have been proposed as the cause of the intensity decay. For example, simulations^17^ showed a progressive self-weaking of TC intensity after reaching the LMI, which is latter confirmed by observations^18^. The changes of coastal sea surface temperature^19^, vertical wind shear^20^ and entrainment of dry air^21–23^ may also modulate the intensity decay from LMI to landfall. From an energetic perspective a TC system is close to the balance of power generation by heat fluxes and surface frictional dissipation when its intensity reaches the LMI^24^. Any subsequent unfavourable environmental perturbation but before landfall may break the balance, reduce the power generation, and therefore lead to an intensity decay dominated by surface friction over oceans.

For the intensity reduction after landfall an empirically exponential decay model has been proposed^25^ and widely used^26–28^. Recently, a physically based algebraic model was also proposed for the decay after landfall^29^. However, there has been a lack of theoretical model for the decay from LMI to LI prior to landfall. In this study we will propose a simple physical model that connects LMI to LI over oceans. We will demonstrate that the intensity decay from LMI to LI can be understood with a physical logistic model.

## Results

The intensity change can be considered as the residual between energy production by ocean enthalpy input and surface dissipation by frictional drag^30^. Our decay model is an extension of the approach proposed by ref^31^. We start with their Eq. (12):

1 $$\frac{\partial {V}_{m}}{\partial t}=\frac{{C}_{D}}{H}{(-{V}_{m}}^{2}+{E{V}_{mpi}}^{2})$$

where *V*_*m*_ is the maximum wind speed near the surface, *V*_*mpi*_ is the maximum potential intensity, *C*_*D*_ is the drag coefficient, *E* is an efficiency taken to be a normalised inertial stability frequency^31^, and *H* is an “effective” depth of vortex. The height parameter *H* can be understood as the depth over which friction acts to spin down the cyclone, which has been shown to be roughly twice the depth of the boundary layer^32^. The first term on the r.h.s of Eq. (1) represents surface dissipation due to friction, and the second term corresponds to energy production.

Since we will apply the model to the intensity decay after LMI that is at least 33 m s^−1^ (i.e., category-1 TCs, see Methods) over a relatively short period of time before landfall (about 1.5 days on average), Eq. (1) can be further simplified to zero order as follows. First, *V*_*mpi*_ during this period may be assumed as stationary since it changes by less than about 5%^33^. Second, the radius of maximum wind (*r*_*m*_) during this short period may be also assumed as stationary for typical change of less than 15%^18^. Third, the Coriolis parameter (*f*) is a small term compared to *V*_*m*_*/r*_*m*_. With these assumptions, the efficiency, *E*, is then proportional to *V*_*m*_ [Eq. (9) in Ref^31^, i.e., $E={\left[(f+2{V}_{m}/{r}_{m})/(f+2{V}_{mpi}/{r}_{mpi})\right]}^{n}$, where *r*_*mpi*_ is the radius of *V*_*mpi*_ and *n* = 1 as recommended^31^], and the second term on the r.h.s. in Eq. (1) can be simplified as *αV*_*m*_, where *α* is a constant for each storm decay. Thus, the simplified version of Eq. (1) can be written in the form of a logistic equation:

2 $$\frac{\partial {V}_{m}}{\partial t}=-\kappa {{V}_{m}}^{2}+\alpha {V}_{m}$$

where $\kappa =\frac{{C}_{D}}{H}$ and $\alpha =\kappa \frac{{r}_{mpi}{V}_{mpi}}{{r}_{m}}$. The parameter *κ* is defined here as a decay parameter. Since the two terms on the r.h.s. represent frictional dissipation and energy production, we have $\kappa >0$ and $\alpha >0$.

Integration from the LMI (*V*_*o*_) at time 0 to an intensity *V*_*m*_ after time *t* during decay yields

3 $$\frac{1}{{V}_{m}}=\frac{1}{{V}_{o}}{e}^{-\alpha t}-\frac{\kappa }{\alpha }({e}^{-\alpha t}-1)$$

Equation (3) is the *logistic decay model* that will be tested against observations.

When $\alpha t$ is small ${e}^{-\alpha t}$ can be approximated to $1-\alpha t$, and therefore Eq. (3) can be simplified to $1/{V}_{m}=1/{V}_{o}+(\kappa -\alpha /{V}_{o})t$. When $\alpha /{V}_{o}\ll \kappa$ as will be shown in Fig. 2, Eq. (3) can be further simplified as:

4 $$\frac{1}{{V}_{m}}=\frac{1}{{V}_{o}} +\kappa t$$

Equation (4) represents an algebraic decay consistent with a previous vortex spin down model^34,35^ when frictional loss dominates. This algebraic decay has been recently validated over land^29^.

For comparison with the logistic decay [Eq. (3)], we also use a simple exponential decay approximation for the intensity decay from LMI to LI over oceans, which can be written as

5 $${V}_{m}={V}_{o}{e}^{-t/\tau }$$

where *τ* can be defined as a decay timescale with a unit of hr^36^.

**Figure 1:**
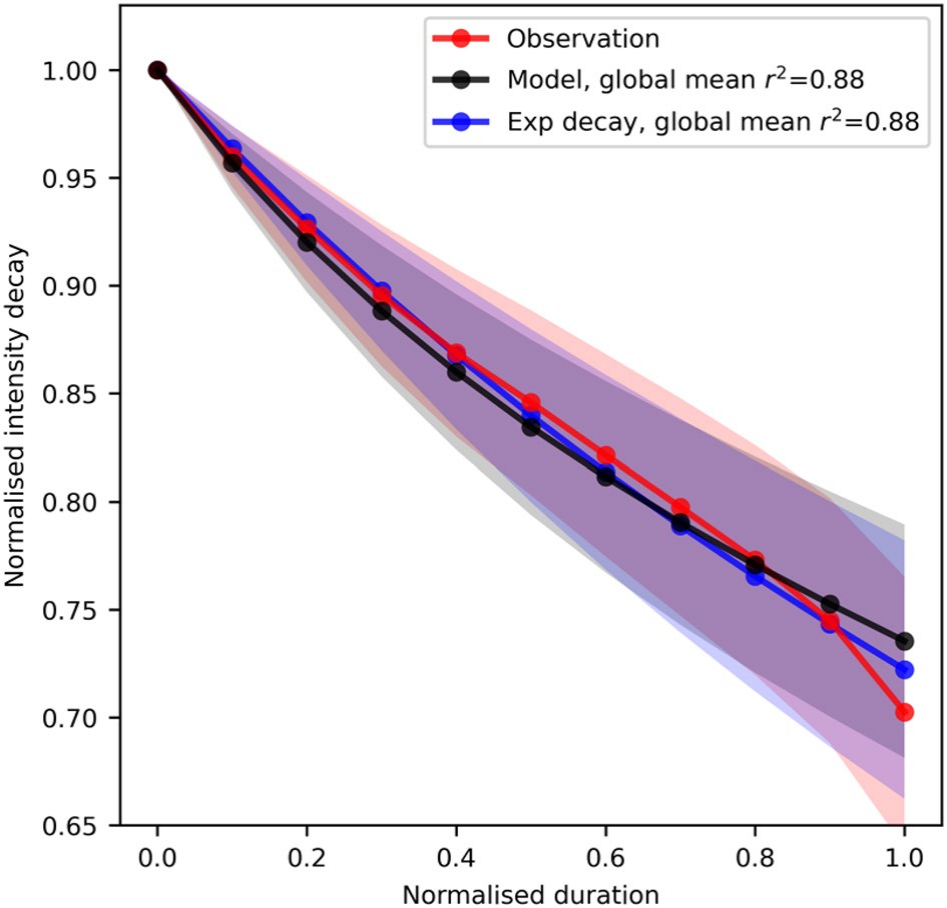
Composite of intensity decay. The observed and fitted intensity decay from LMI to LI. The algebraic decay model [Eq. (3)] and an exponential decay [Eq. (5)] are used for the fit. Prior to compositing all TCs, each time series is normalised by its LMI and then interpolated onto the deciles of its duration. The shadings show one standard error of the mean at each decile point. Normalised duration time 0 and 1 represent the times of LMI and LI. The coefficient of determination, *r*^2^, are given in the legend for global TCs.

Figure 1 shows that the observed intensity decays to the LI on average by about 25% of LMI. The decay time is defined from the last LMI to landfall. Due to the temporally discrete best-track records, the last TC centre record is over land. The observed intensity in Fig. 1 therefore decays more rapidly in the last 10% of decay duration, which reflects an abrupt enhancement of surface friction after landfall. The fit of the logistic decay model, i.e., Eq. (3), shows an excellent ability to quantitatively characterise the observed intensity decay from LMI to landfall with a mean coefficient of determination (*r*^2^) of 0.88 (Fig. 1). The same mean *r*^2^ is obtained if the exponential decay, i.e., Eq. (5), is used for the fit. The observed mean LI, LMI and decay duration of global TCs are 38 m s^−1^, 54 m s^−1^ and 35 h, respectively (Fig. S1). The fitted mean *κ*, *α* and *τ* are 2.2 × 10^–7^ m^−1^, 3.1 × 10^–6^ s^−1^ and 92 h, respectively (Fig. S1).

**Figure 2:**
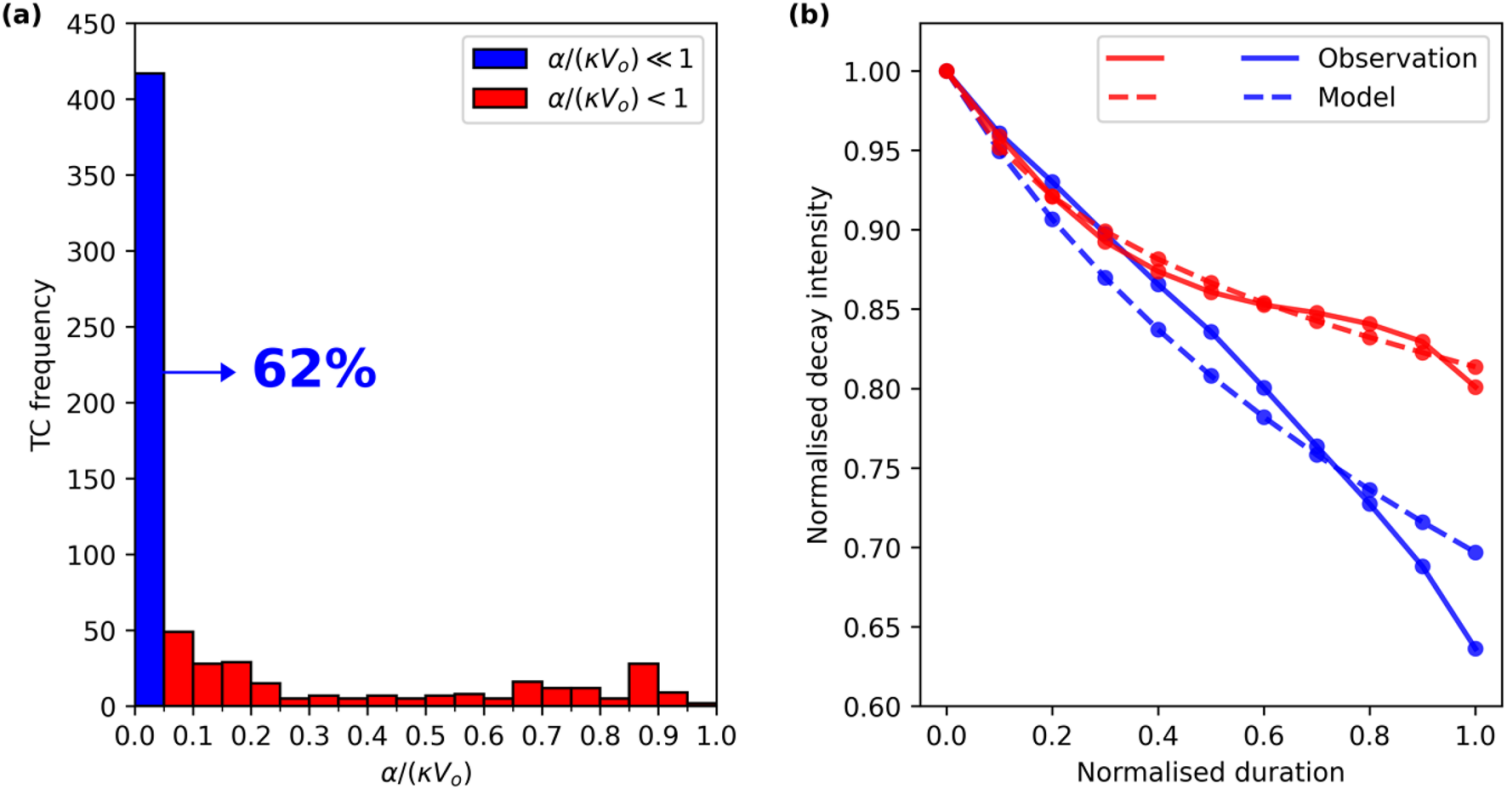
Comparison between frictional dissipation and energy production [$\alpha /(\kappa {V}_{o})$]. (**a**) Histogram of $\alpha /(\kappa {V}_{o})$ with a bin width of 0.05. (**b**) As in Fig. 1, but for the two groups with $\alpha /(\kappa {V}_{o})\ll 1$ (blue) and $\alpha /(\kappa {V}_{o})<1$ (red).

With the fitted *κ* and *α*, the relative importance of frictional dissipation ($\kappa {{V}_{m}}^{2}$) and energy production ($\alpha {V}_{m}$) in Eq. (2) is compared in terms of their ratio at LMI [$\alpha /(\kappa {V}_{o})$]. Since here we focus on the decay of intensity, by definition, dissipation is larger than production, i.e., all $\alpha /(\kappa {V}_{o})<1$. For about 60% of decaying TCs, the energy production term is much less than 5% of the frictional dissipation term (the blue bar in Fig. 2a) and thus is negligible. Figure 2b shows that when the production term is important (although still less than the dissipation term), the logistical model performs very well (red lines), which gives confidence in the physical assumptions of the model.

**Figure 3:**
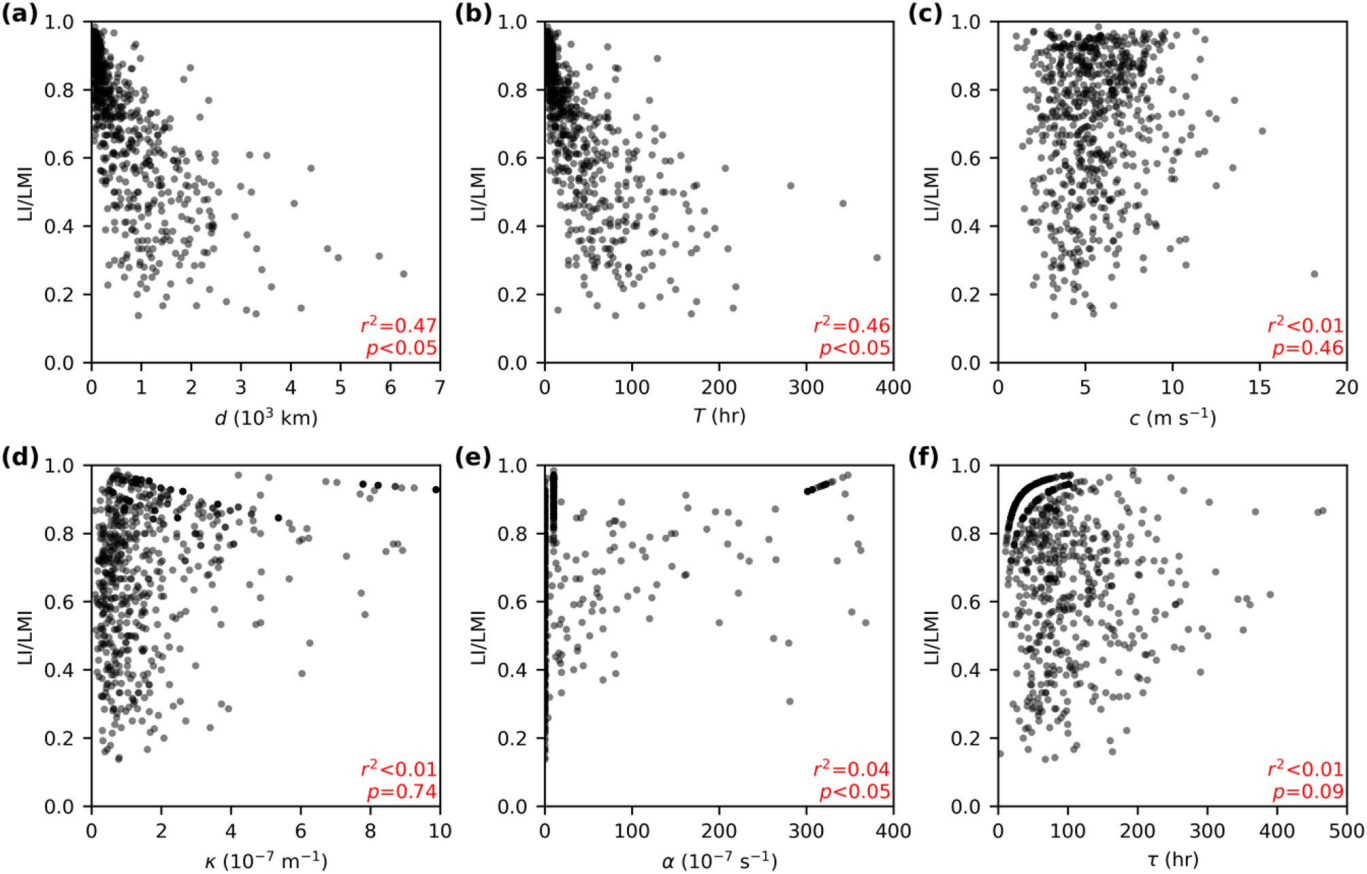
Intensity reduction and controlling factors. The fractional intensity reduction (LI/LMI) against: (**a**) total distance, *d*, from LMI to landfall; (**b**) decay duration, *T*, to landfall (hours); (**c**) mean translation speed, *c*, during decay, (**d**) model parameter *κ* in a logistic decay; (**e**) as in (**d**) but for *α*, (**f**) decay timescale, *τ*, in an exponential decay. The coefficient of determination, *r*^2^, is given in the bottom-right corner. Two-sided *p*-value is estimated using Wald test with *t*-distribution of the test statistic.

The landfall intensity, LI, can be understood with the logistic decay model by rewriting Eq. (3) as

6 $$\frac{1}{LI}=\frac{1}{LMI}{e}^{-\alpha T}-\frac{\kappa }{\alpha }({e}^{-\alpha T}-1)$$

where *T* is the duration of the total decay from an initial *V*_*o*_ of LMI to LI, which can be further written as *d*/*c*, where *d* is the distance travelled from LMI to landfall and *c* is the mean translation speed of the TC during decay. Figure 3 shows that the relative intensity reduction, defined as the ratio of LI to LMI, is strongly and significantly correlated with the distance, *d*, travelled during the decay (*r*^2^ = 0.47, *p* < 0.05, Fig. 3a), and the decay duration, *T* (*r*^2^ = 0.46, *p* < 0.05, Fig. 3b). There is also a significant relationship between *d* and *T* themselves (*r*^2^ = 0.57, *p* < 0.05). The translation speed, *c* (i.e., *d*/*T* for each TC), however, appears to have no significant impact on the decay (*p* = 0.46, Fig. 3c). It is important to note that the strong correlation between intensity decay and decay distance *d* (and therefore decay duration *T*) is independent of the choice of decay models. By contrast, the fitted parameters (see Methods), i.e., *κ* and *α* in the logistic model and *τ* in the exponential decay, show surprisingly weak correlation with the relative intensity decay from LMI to LI (*r*^2^ < 0.05, Fig. 3d–f). For the TCs with major LIs (Fig. S2), the distance *d* and duration *T* also show stronger correlations with the decay than the other parameters examined.

**Figure 4:**
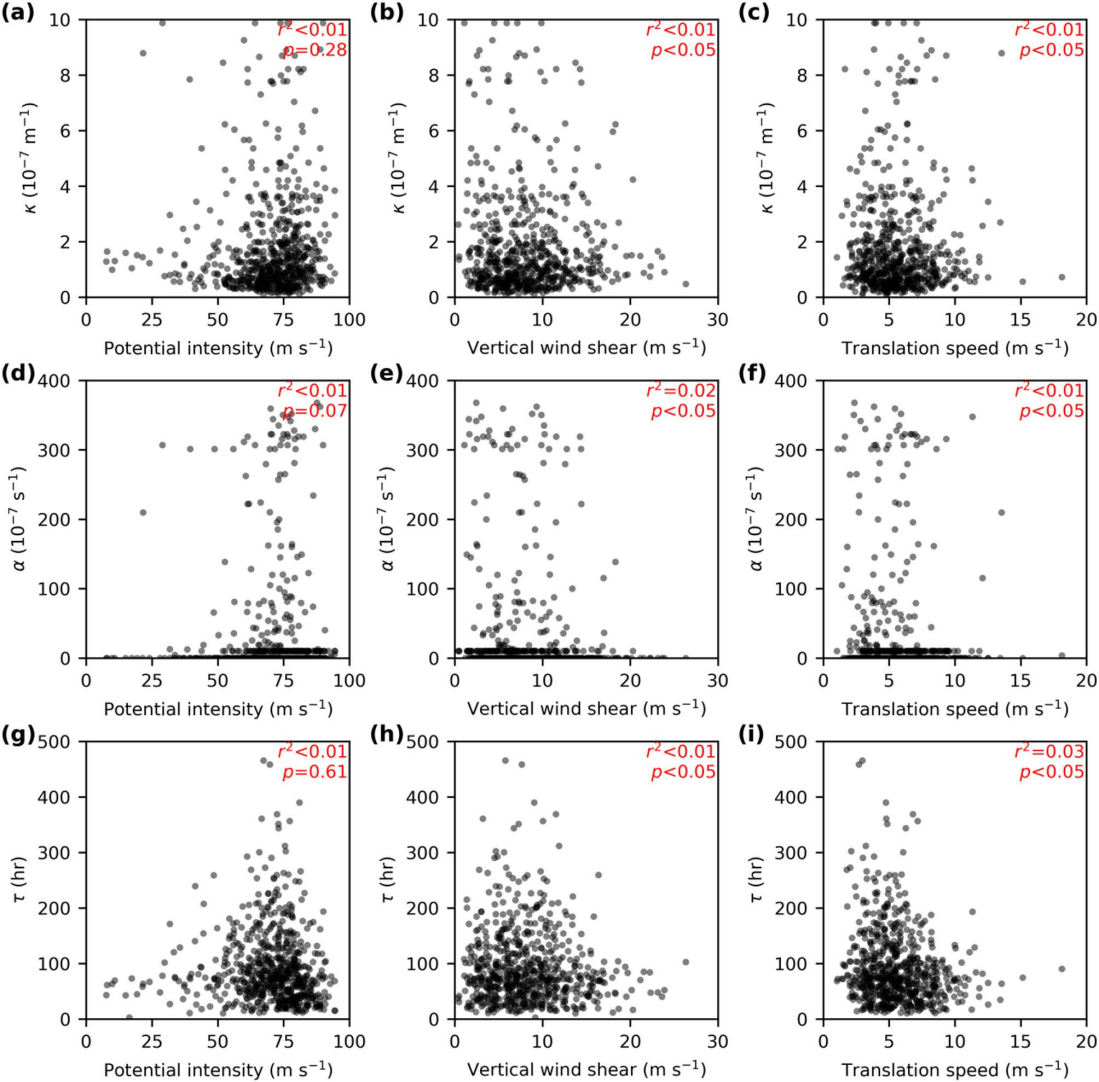
Fitted parameters and environmental conditions. (**a**–**c**) Decay parameter *κ* in the logistic decay model. (**e**–**f**) As in (**a**–**c**), but for *α*. (**g**–**i**) Decay timescale *τ* in an exponential decay. Three environmental conditions are (**a**, **d**, **g**) the potential intensity, (**b**, **e**, **h**) vertical wind shear, and (**c**, **f**, **i**) steering flow approximated by the TC translation speed.

We next examine the potential environmental control of the intensity decay parameters. Here three mean environmental conditions (see Methods) during decay are analysed: the potential intensity, vertical wind shear and steering flow approximated by TC translation speed. Figure 4 shows that none of the decay parameters is significantly associated with any of the environmental conditions. Similar results are found (not shown) for the TCs with the distance from LMI to LI above or below the median distance (500 km). The location of TC activity in the western North Pacific can be modulated by the El Niño-Southern Oscillation^47^. However, the detrended annual mean distance, *d*, in the northern West Pacific and the Niño3.4 index (August-October) are not significantly correlated (*r*^2^ = 0.1, *p *value = 0.06). The analysis in Figs. 3 and 4 points to the dominant role of the variability of *d*, rather than the fitted *κ*, *α* and *τ*, or other environmental conditions, as the primary cause of the LI variability.

The potential damage caused by TC landfall increases substantially for major TCs^37^. We next focus on the properties of these storms with major LIs (≥ 50 m s^−1^) in terms of the variables in the decay models. As expected, very high LMIs are found in these cases. The global median LMI of these TCs is ranked at about the 80th percentile of the LMIs of all the landfalling TCs (Table 1), which shows the connection between extreme LMIs and major LIs. Globally, relatively small distance (short duration) is important to achieving a weak decay from LMI to LI and hence high LI. The LMI of the TCs with major LIs occurs very close to land (122 km), less than half of the median distance to land for all global landfalling cases (282 km). However, the translation speed, *c*, is not unusual. All the variables in Table 1 as mentioned above are independent of decay approximations (logistic or exponential). Regarding the fitted parameters in two decay models, *τ* and *α* of the TCs with major LIs are close to the medians of all landfall TCs. The energy production term is therefore not unusual for these cases. Interestingly, a small *κ* appears to contribute to a high LI, which will be discussed in the next section.

**Table 1:** Percentiles of the median of the decay-model variables of the TCs with major landfalls (LI ≥ 50 m s^−1^) ranked in all the global landfall TCs (LMI ≥ 33 m s^−1^).

	LMI	d	T	c	κ	α	τ
Percentile	80 ± 3	32 ± 4	39 ± 3	54 ± 5	33 ± 3	56 ± 6	50 ± 5

The standard deviation of the percentiles is also estimated (given after “ ± ”) with bootstrapping. For each variable examined, the TCs with major landfalls are resampled for 10,000 times with replacements and a distribution of the percentiles of the median is then obtained (with 10,000 samples). The standard deviation is calculated with the resampled percentile distribution.

## Discussion and conclusions

The logistic intensity decay, i.e., Eq. (2), is a modification of an intensification theory^31^. Two assumptions based on observations^18,33^ are applied to derive the decay model, that is, the *V*_*mpi*_ and *r*_*m*_ are stationary to first approximation from the LMI to LI. Since the derived model captures the intensity decay over oceans as shown in Fig. 1, these two zero-order assumptions give plausible results. A similar logistic intensification model has been used in operational forecast^27^, but developed from a statistical point of view. This study shows the validity of the logistical model for decay, but from a theoretical perspective.

Previous study^38^ points to the importance of ventilation of the boundary layer mass by deep convection. Once this ventilation ceases to be sufficient then the spin down mechanism will dominate. From an energetic perspective, TC intensification and decay can be understood as a competition between power production from ocean enthalpy input and surface frictional dissipation^31^. These two factors reach a balance at the time of LMI^24^. However, it has been found that even at the time of LMI the surface frictional dissipation rate at the radius of maximum wind is about 25% larger than the local energy production rate^39^. Thus, an intensity decay can be easily triggered post LMI with minor environmental influence if the excess frictional dissipation under the eyewall cannot be balanced by the energy production outside the eyewall in time, or the boundary layer ventilation by convection becomes ineffective. Our analysis shows that the decay from LMI to landfall can be mostly captured by frictional dissipation in about 60% of the TCs without the need to invoke a strong role for energy production by enthalpy fluxes.

The simplified algebraic decay [Eq. (4)] also predicts that the LI can be understood with two terms: an upper limit, 1/LMI, and the decay, *κT*. In line with this prediction, Table 1 suggests that TCs with major LI usually have relatively small *κ*. The median *κT* of the TCs with major LI is ranked at the 18th percentile of that for all landfall TCs, which is a more extreme percentile compared to the rankings in Table 1. It is nevertheless surprising that in general, the decay parameter, *κ*, does not have a strong predictive relationship with the intensity reduction when all landfall TCs are included in the statistics. We note that there may be some cancellation effects as *κ* depends on both the effective vortex depth *H* and the drag coefficient *C*_*D*_ which may vary in the same direction when TC is relatively weak. However, there are no direct observations of either term. There are also no systematically significant relationships between *κ* and either environmental thermodynamic condition or vertical wind shear. Although *κ* appears on average to play a minor role in the climatology of the decay, we still expect environmental conditions to be important for individual cases^40^ and some subgroups of TCs, for example, those experiencing a rapid weakening^41^. We show that a small *κ* is a necessary condition for major TCs at landfall.

The decay parameter, *κ*, can be quantitatively broken into physically relevant components: the ratio of surface drag coefficient to an effective depth of vortex (i.e., *κ* = *C*_*D*_*/H* as defined in section “Results”). The mean fitted *κ* of 2.2 × 10^–7^ m^−1^ is also consistent with typical values of *C*_*D*_ = 1.0 × 10^–3^ over oceans^42^ and an *H* = 4.5 km. Recall that *H* is defined as the “effective” vortex depth and is thus also the depth over which friction acts to spin down the cyclone. This estimation of 4.5 km is close to a boundary layer depth scale of 5 km in the eyewall as previously hypothesised^32^. We also note that it is approximately the height below which the buoyancy and Richardson number decrease rapidly in model simulations^43^.

We may also define a half-life of intensity decay with the simplified algebraic model as 1/(*κ*LMI). This definition of half-life shows a self-regulating nature of intensity decay, that is, a more intense TC decays faster, whereas an exponential decay depends solely on the decay timescale *τ*^36^. Previous study^28^ found that the exponential decay approximation does not perform well when the initial decay intensity is high, which supports our finding here that the intensity decay should depend not only on the decay parameter (i.e., *κ* or *τ*), but also the initial decay intensity. This dependence on the initial decay intensity can lead to dampening of any positive LMI trend at landfall through an increasingly more rapid decay. For example, according to Eq. (4) the intensity difference of two TCs with LMIs of 70 and 50 m s^−1^ converges at landfall to a difference of only 5 m s^−1^ for *T* = 48 h and *κ* = 1 × 10^–7^ m^−1^. This may provide an explanation for the less significant LI trend^44^ compared to the LMI trend over oceans^4,45,46^, and fundamentally distinguishes the proposed physically based logistic model from exponential decay models^25,26^.

Major TCs at landfall are characterised by both high LMI and close proximity to land. The proposed decay model usefully points to the stringent and rare conditions required for a TC to make landfall as a major TC. Most climate models (e.g., Ref^47^) already struggle to generate the most intense storms in terms of LMI. This importance of distance creates another challenge to simulate plausible LIs given the model typical horizontal resolution of more than 100 km. Our findings may also have implications for weather forecasting and stochastic catastrophe models used in the insurance sector. Forecasting the most damaging storms with extreme intensities at landfall requires forecasts of both the LMI and its location close to land. For the stochastic models the TC evolution from genesis to landfall may be much less important than the ability to modelling the LMI, LMI location and subsequent decay. There has been much discussion in the literature and in the public domain on the role of global warming in changing TC intensity. Thermodynamic arguments point to the role of a warming ocean as the source of heat to drive intensity changes. Here we show that to understand the changes at landfall this is only part of the story. The changes in distance from LMI to landfall are also of critical importance to understand the past variability and predict future changes in tropical cyclone intensities at landfall.

## Methods

### Data

We consider landfalling TCs with an LMI of at least hurricane-force wind (LMI ≥ 33 m s^−1^) for the period 1982–2019. In this period we have the highest confidence in data quality and the completeness of global TC observations^48^. We take the TC best-track data from the International Best Track Archive for Climate Stewardship (IBTrACS) v04r00^49^, with the original data sources from the National Hurricane Center and the Joint Typhoon Warming Center. The TC positions and the other measures in the IBTrACS are interpolated from 6-h to 3-h intervals with splines and linear interpolations, respectively. In this study we use the IBTrACS best-track data at 3-h intervals, i.e., 00, 03, 06, 09, 12, 15, 18 and 21 Universal Time Coordinates. Only TCs within the 40^o^N/S latitude band are considered to reduce extra-tropical impacts.

### Landfall criterion and decay period

The distance to the nearest land is available in the IBTrACS for each TC location. The smallest landmass used for the distance calculation is 1400 km^2^, equivalent to the area of Kauai, Hawaii. This distance is positive when the TC centre is over the ocean and drops to zero after landfall. We define a landfall at the time when the distance to land is reduced to zero from a positive distance. Some TCs made landfall more than once. In these cases, only the landfall with the highest landfall intensity is considered. A TC may also reach the same LMI more than once during its lifecycle. We define the decay period from the time of the last LMI to landfall. Seven percent of global landfalls happen at LMI, in which case the LMI and LI are equal and there is no intensity decay.

### Parameter estimation

The parameters in the decay models [i.e., *κ* and *α* in Eq. (3) and *τ* in Eq. (5)] are estimated with the least-squares minimization fit to the entire intensity evolution from LMI to LI for each TC. There are on average 13 data points including LI and LMI for all the landfalling TCs with LMI ≥ 33 m s^−1^, and 6 data points for the TCs with major LI (LI ≥ 50 m s^−1^). For each fit we use the observed LMI for *V*_*o*_ and decay duration for *t*.

### Environmental conditions

The hourly atmospheric reanalysis product, ERA5^50^, is used to calculate the actual TC-related vertical wind shear and maximum potential intensity (MPI) for each TC during the period 1982–2019 rather than monthly means. For each TC the variables are calculated three-hourly along its track during decay, and we then take the mean to represent this environmental condition for the TC. Vertical wind shear is calculated as the magnitude of wind vector difference between 200- and 850-hPa pressure levels in the 2-to-8-degree-latitude annulus around TC centres. The MPI^1^ is calculated along the track three days before a TC arrives.

## Supplementary Information

Supplementary Information.

## Data Availability

Tropical cyclone best track data can be downloaded from the National Centers for Environmental Information website (https://www.ncei.noaa.gov/data/international-best-track-archive-for-climate-stewardship-ibtracs/v04r00/access/csv/ibtracs.ALL.list.v04r00.csv). The ERA5 reanalysis data is available at the European Centre for Medium-Range Weather Forecasts (https://cds.climate.copernicus.eu/cdsapp#!/dataset/reanalysis-era5-pressure-levels?tab=form).

## Code availability

All codes used to analyse and plot the data are available at https://data.hpc.imperial.ac.uk/resolve/?doi=8721&access= .
