# Author Correction: On the intensity decay of tropical cyclones before landfall

**DOI:** 10.1038/s41598-022-17834-4

**Published:** 2022-08-04

**Authors:** S. Wang, R. Toumi

**Affiliations:** Department of Physics, Imperial College London, London, SW7 2AZ UK

Correction to: *Scientific Reports* 10.1038/s41598-022-07310-4, published online 28 February 2022

The original version of this Article contained an error in the Results section below Equation 2, where the Greek letter κ was omitted in the explanation of variable α:

“where $$\kappa\;=\frac{{C}_{D}}{H}\, {\mathrm{and }}\, \alpha = \frac{{r}_{mpi}{V}_{mpi}}{{r}_{m}}$$ ”

now reads:

“where $$\kappa\;=\frac{{C}_{D}}{H}\, {\mathrm{and }}\, \alpha = \kappa \frac{{r}_{mpi}{V}_{mpi}}{{r}_{m}}$$ ”

The original version of this Article has been corrected.

