# Supplementary material for: On the intensity decay of tropical cyclones before landfall
Source: Sci Rep. 2022 Feb 28;12:3288. doi: 10.1038/s41598-022-07310-4 (PMC8885634; doi:10.1038/s41598-022-07310-4)
Supplement: Supplementary file 1 — Supplementary Information. [file 41598_2022_7310_MOESM1_ESM.docx]

**Supplementary Materials for**

**On the intensity decay of tropical cyclones before landfall**

S. Wang^1*^ and R. Toumi^1^

^1^Department of physics, Imperial College London, London, SW7 2AZ, UK

*Correspondence to: [shuai.wang@imperial.ac.uk](mailto:shuai.wang@imperial.ac.uk)

**This PDF file includes:**

Fig. S1-S2


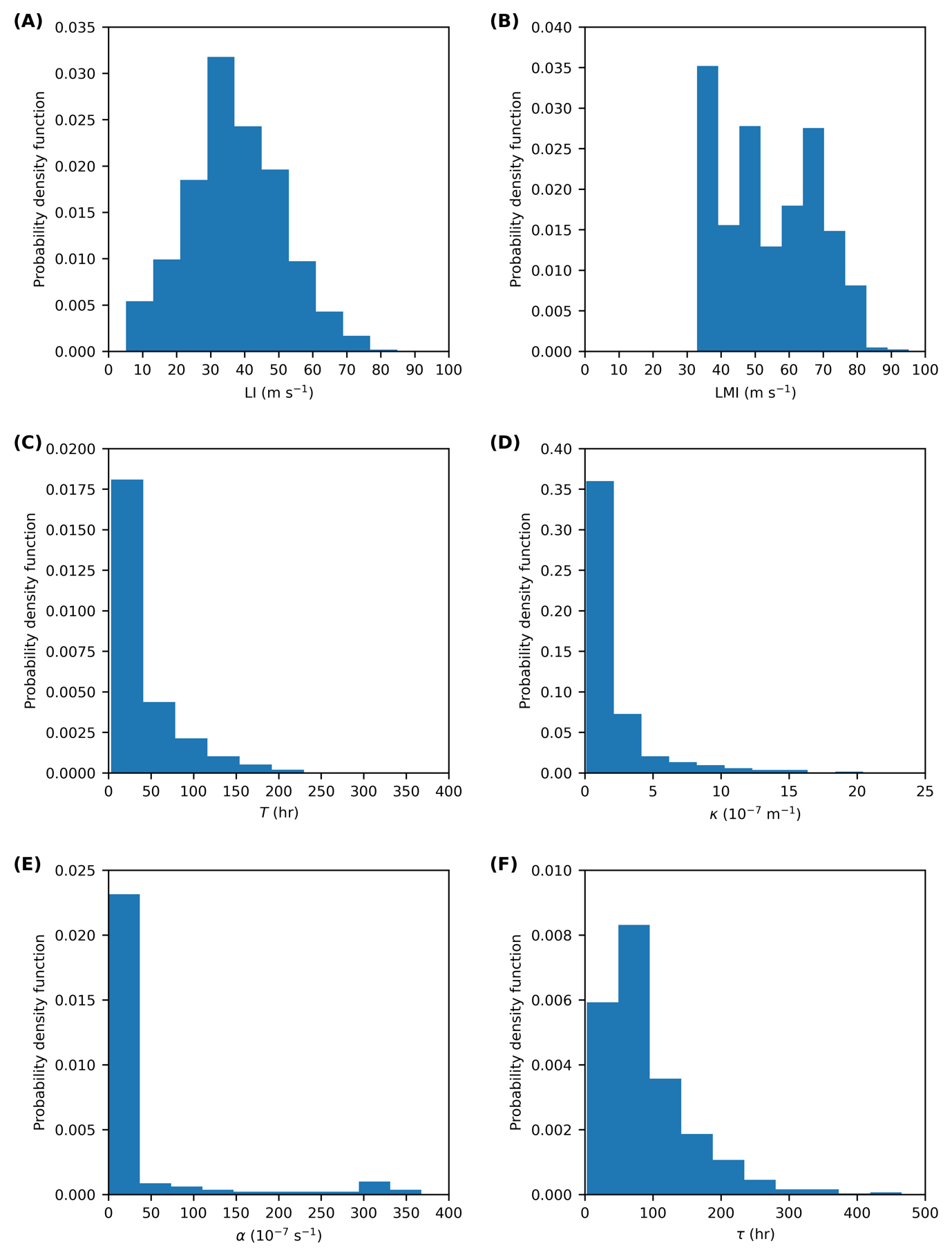


Fig. S1. Probability density function of LI, LMI, *T*, *κ*, *α* and *τ* of global landfall TCs (LMI≥33 m s^-1^).


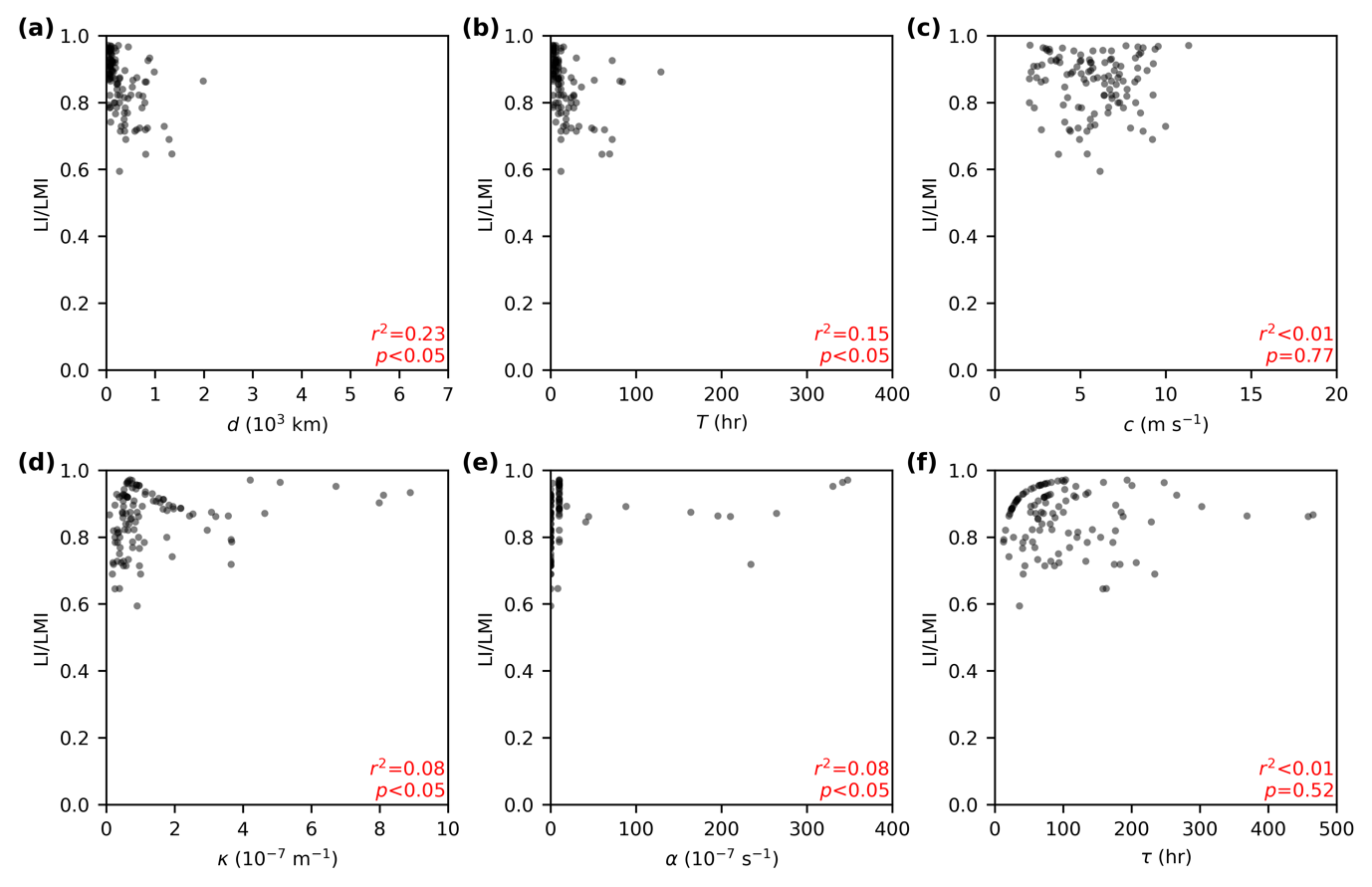


Fig. S2. As in Fig. 3, but for TCs with major LIs.
